# Peroxisome Proliferator-Activated Receptor Gamma Coactivator-1-Alpha in Endometriosis: Expression, Regulation, and Potential Role

**DOI:** 10.7759/cureus.77341

**Published:** 2025-01-12

**Authors:** Chunlong Han, Jie Chen

**Affiliations:** 1 Obstetrics and Gynecology, Affiliated People's Hospital of Fujian University of Traditional Chinese Medicine, Fuzhou, CHN

**Keywords:** caspase-3, caspase-9, endometriosis, errα, erβ, il-6, pgc-1α

## Abstract

Objective: The present study was designed to comprehensively analyze the expression profiles of peroxisome proliferator-activated receptor-γ coactivator-1α (PGC-1α), estrogen-related receptor-α (ERRα), estrogen receptor-β (ERβ), interleukin-6 (IL-6), cysteinyl-aspartic acid-specific protease-3 (caspase-3), and cysteinyl-aspartic acid-specific protease-9 (caspase-9) in endometriosis tissues. It also aimed to elucidate the hitherto unclarified role of PGC-1α in the processes of proliferation, apoptosis, and gene expression regulation of human endometrial stromal cells, thereby providing novel insights and identifying potential molecular targets for advancing endometriosis treatment modalities.

Methods: A total of 49 ectopic endometrial tissue samples and 50 normal endometrial tissue samples were meticulously collected from patients who underwent gynecological surgeries in the People's Hospital Affiliated to Fujian University of Traditional Chinese Medicine in Fuzhou, China, between January 2022 and January 2023. Immunohistochemistry was used to detect protein expression levels. Human primary endometrial stromal cells were transfected and grouped for in-depth analysis. Real-time quantitative polymerase chain reaction (RT-qPCR) was utilized to measure gene expression, the cell counting kit 8 (CCK-8) assay was employed to evaluate cell proliferation, and the lactate dehydrogenase (LDH) method was used to determine cell death.

Results: In endometriosis tissues, compared with normal endometrial tissues, the expression levels of PGC-1α, ERRα, and IL-6 were significantly increased. The expression of ERβ was also elevated, while the expression levels of caspase-3 and caspase-9 were decreased. In cell experiments, after transfection with interference plasmids to reduce PGC-1α expression, the expression levels of ERRα and ERβ were decreased, the expression of IL-6 was increased, and the expression levels of caspase-3 and caspase-9 were augmented. Conversely, after transfection with overexpression plasmids to enhance PGC-1α expression, the expression levels of ERRα and ERβ were elevated, the expression of IL-6 was diminished, and the expression levels of caspase-3 and caspase-9 were decreased. Moreover, when PGC-1α expression was interfered with by siRNA, cell proliferation was attenuated (albeit not statistically significant), and cell apoptosis was enhanced. Overexpression of PGC-1α promoted cell proliferation and inhibited cell apoptosis.

Conclusion: PGC-1α potentially plays a crucial role in the pathogenesis of endometriosis by regulating ERRα, ERβ, IL-6, and apoptosis-related factors. This study provides a strong theoretical foundation and a novel direction for developing endometriosis treatment strategies. To the best of our knowledge, it is the first to systematically explore the relationship between PGC-1α and these key factors in endometriosis, filling a knowledge gap. Future studies are needed to further dissect its mechanism and realize its clinical application potential.

## Introduction

With an incidence rate usually between 10% and 15%, endometriosis, a common gynecological condition that primarily affects women in the reproductive age range, is primarily defined by the presence of active endometrium outside the uterine cavity [1]. Infertility, increasing dysmenorrhea, and persistent pelvic pain are among the crippling symptoms that patients frequently experience. Sufferers' total quality of life significantly declines as a result of these symptoms, which also have a negative psychological impact in addition to their physical health.

The three primary treatment modalities for endometriosis in the contemporary clinical setting are surgery, medication, and a combination of the two [2]. Fertility may be negatively impacted by surgical techniques that attempt to remove or ablate the ectopic endometrial tissue, but they also involve the risk of recurrence and possible iatrogenic injury to the reproductive organs [3]. Although pharmacological treatment works by adjusting hormone levels to inhibit the growth of endometrial tissue, prolonged use can result in a number of negative side effects, including mood swings, vasomotor symptoms, and bone loss, and the rate of recurrence following drug discontinuation is still quite high [4]. Therefore, the main goals and challenges of modern gynecological research are a more thorough investigation of the pathophysiology and the search for more effective treatment targets and tactics.

Clarifying the role that intracellular molecular signaling pathways play in the pathophysiology of endometriosis has been the focus of much research in recent years. The regulation of cellular energy metabolism by peroxisome proliferator-activated receptor-γ coactivator-1α (PGC-1α) has become a major area of interest [5]. PGC-1α is essential for oxidative metabolism, energy balance, and mitochondrial biogenesis. There is mounting evidence that PGC-1α and endometriosis pathophysiology may be related in gynecology [6]. Nevertheless, nothing is known about the exact mechanism of PGC-1α in endometriosis.

In order to better understand the effects of PGC-1α on the proliferation, apoptosis, and gene expression profiles of human endometrial stromal cells, this study aims to perform a more thorough and in-depth analysis of the expression patterns of PGC-1α and its related signaling molecules (estrogen-related receptor-α (ERRα), estrogen receptor-β (ERβ), interleukin-6 (IL-6), cysteinyl-aspartic acid-specific protease-3 (caspase-3), and cysteinyl-aspartic acid-specific protease-9 (caspase-9)) in endometriosis tissues. By fulfilling these objectives, it is anticipated that this study will provide fresh perspectives and a strong theoretical basis for comprehending the intricate pathophysiology of endometriosis, opening the door for the creation of creative therapeutic approaches and the discovery of possible therapeutic targets.

## Materials and methods

Clinical data

Between January 2022 and January 2023, we gathered 50 cases of normal endometrial tissue and 49 cases of endometriotic tissue from patients undergoing surgery at the Affiliated People's Hospital of Fujian University of Traditional Chinese Medicine in Fuzhou, China. The inclusion criteria for patients in this study were as follows: for the endometriosis group, women aged between 18 and 45 years who were diagnosed with endometriosis through laparoscopic or laparotomy surgery and had a confirmed histological diagnosis; for the control group (providing normal endometrial tissue), women who underwent hysterectomy for non-endometriosis-related reasons, such as uterine fibroids or prolapse. Exclusion criteria included patients with other gynecological malignancies, severe comorbidities (such as heart disease, liver disease, or kidney disease), or those who had received hormonal treatment within the past three months before surgery.

For the collection of normal endometrial tissue, the endometrial tissue was collected from the uterine cavity during the surgery and immediately snap-frozen in liquid nitrogen or fixed in formalin for further analysis. For the collection of endometriotic tissue, samples were taken from the ectopic endometrial lesions during laparoscopic or laparotomy procedures. The anatomical sites of the endometriotic tissue included the ovaries, fallopian tubes, pelvic peritoneum, and other pelvic organs.

The case group's body mass index (BMI) was 21.72 ± 2.31 kg/m², and their average age was 33.05 ± 4.06 years. With a BMI of 21.26 ± 3.18 kg/m² and an average age of 32.44 ± 4.99 years, the control group did not differ significantly from the other groups (P > 0.05). A pathologist diagnosed every material. The study was approved by the Ethics Committee of the Affiliated People's Hospital of Fujian University of Traditional Chinese Medicine, and all patients provided informed consent (approval number: 2021-050-02).

Main reagents

The primary antibodies used included rabbit anti-human antibodies against PGC-1α, ERRα, ERβ, IL-6, caspase-3, and caspase-9, obtained from Beijing Boaosen Biological Technology Co., Ltd. (Beijing, China). The two-step immunohistochemistry kit and 3,3′-diaminobenzidine (DAB) kit were purchased from Beijing Zhongsheng Jinqiao Biotechnology Co., Ltd. (Beijing, China). The cell counting kit 8 (CCK-8) kit was sourced from Changsha Aibwei Biotechnology Co., Ltd. (Changsha City, China). The lactate dehydrogenase (LDH) test kit was purchased from Jiangsu Kaiji Biotechnology Co., Ltd. (Beijing, China). Lipofectamine 2000 transfection reagent was obtained from Thermo Fisher Scientific (Waltham, MA).

Immunohistochemistry

Tissue specimens were processed into 3 μm-thick sections, which were then deparaffinized, hydrated, and washed. Antigen retrieval was carried out using citrate buffer, followed by cooling and washing. Endogenous peroxidase was inactivated, and primary antibodies were incubated overnight at 4°C, followed by a 30-minute incubation with secondary antibodies at 37°C. After washing, the DAB chromogenic reaction was conducted, excess chromogen was washed off, and the sections were counterstained with hematoxylin, dehydrated, and mounted. Observations were made under a microscope at 100x magnification, and optical density (OD) values were analyzed using ImageJ software (National Institutes of Health, Bethesda, MD).

Cell transfection

Human primary endometrial stromal cells (purchased from Wuhan Puno Sai Life Technology Co., Ltd. (Wuhan, China)) were digested with trypsin, centrifuged, and divided into five groups: (1) The control group received no treatment; (2) The sh-NC group was transfected with the empty interference plasmid; (3) The sh-PGC-1α group was transfected with the PGC-1α interference plasmid; (4) The LV-NC group was transfected with the empty overexpression plasmid; (5) The LV-PGC-1α group was transfected with the PGC-1α overexpression plasmid. Each group had three replicates. Lip2000 and plasmids were mixed in sterile centrifuge tubes in accordance with the manufacturer's instructions.

The CCK-8 assay

After 48 hours of transfection, CCK-8 assays were performed according to the manufacturer's instructions, with three replicates per group. Absorbance (OD) values were measured at 450 nm using a microplate reader, and averages were calculated for plotting.

Lactate dehydrogenase detection

After 48 hours of transfection, the supernatants of the above groups were collected, and 10 μL was added to the test kit according to the manufacturer's instructions. The mixture was homogenized and incubated at room temperature for three minutes. Absorbance was measured at 440 nm with distilled water as the blank.

Real-time quantitative polymerase chain reaction (RT-qPCR) detection

Total RNA was extracted using the Trizol method, and messenger ribonucleic acid (mRNA) was reverse transcribed into complementary DNA (cDNA). Specific gene sequences were searched in the National Center for Biotechnology Information (NCBI) database, and primers were designed using Primer5 and synthesized by Beijing Qingke Biotechnology Co., Ltd. (Beijing, China). The PCR reaction mixtures were prepared, including reverse transcription products, primers, water, and SYBR Green PCR Master Mix (Sigma Aldrich, St. Louis, MO). The quantitative PCR program was set as follows: 95°C for 10 minutes, 95°C for 15 seconds, and 60°C for 30 seconds, followed by melt curve analysis from 60°C to 95°C. The primer sequences are listed in Table 1.

**Table 1:** Primer list H-GAPDH: Human glyceraldehyde-3-phosphate dehydrogenase; H-ERβ: human estrogen receptor β; H-ERRα: human estrogen-related receptor α; H-IL6: human interleukin-6; H-CASP3: human cysteinyl aspartate specific proteinase-3; H-CASP9: human cysteinyl aspartate specific proteinase- 9

Name	Sequence	Length
H-GAPDH	Forward (F): CACAGCCTCAAGATCATCAGC Reverse (R): GGTCATGAGTCCTTCCACGAT	104bp
H-ERβ	Forward (F): GCAATAACCACCCCTGACCCAA Reverse (R): TACGCATTTCCCCTCATCCCT	149bp
H-ERRα	Forward (F): TCGGACTGGTCAGGGTTCAG Reverse (R): AGCCCCTTCCCAGACATAGA	213bp
H-IL6	Forward (F): GCAATAACCACCCCTGACCCAA Reverse (R): GCTACATTTGCCGAAGAGCC	154bp
H-CASP3	Forward (F): TGGCAACAGAATTTGAGTCCT Reverse (R): ACCATCTTCTCACTTGGCAT	161bp
H-CASP9	Forward (F): AAGCCAACCCTAGAAAACCTTACCC Reverse (R): AGCACCGACATCACCAAATCCTC	126bp

Statistical analysis

Data were analyzed using Prism software, version 9.50 (GraphPad Software, La Jolla, CA). Count data were presented as counts or percentages, and normally distributed continuous data were expressed as the mean ± standard deviation. Comparisons between the two groups were conducted using t-tests, with P < 0.05 regarded as statistically significant.

## Results

Expression of PGC-1α, ERRα, ERβ, IL-6, caspase-3, and caspase-9 in endometriotic and normal endometrial tissues

**Figure 1:**
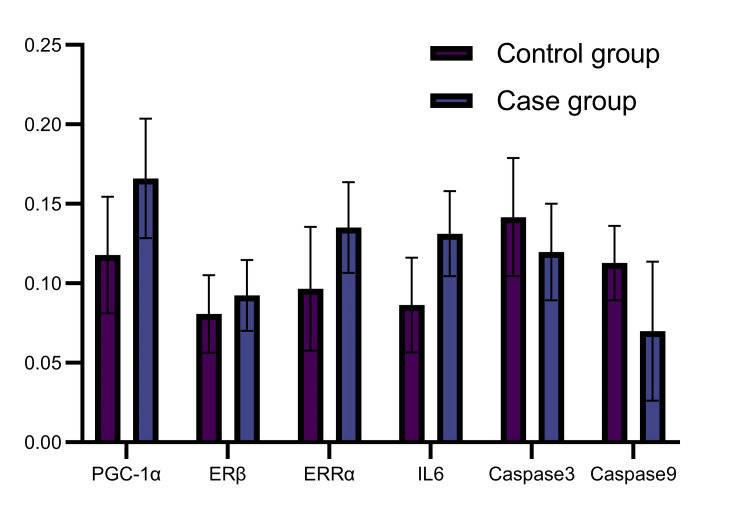
Comparison of the expression of PGC-1α, ERRα, ERβ, IL-6, caspase-3, and caspase-9 in endometriotic and normal endometrial tissues PGC-1α: profiles of peroxisome proliferator-activated receptor-γ coactivator-1α; ERRα: estrogen-related receptor-α; ERβ: estrogen receptor-β; IL-6: interleukin-6; caspase-3: cysteinyl-aspartic acid-specific protease-3; caspase-9: cysteinyl-aspartic acid-specific protease-9

There was a significant difference (P < 0.0001) between the OD values of PGC-1α in endometriotic tissues (0.1178 ± 0.0366) and normal endometrial tissues (0.1660 ± 0.0376). In endometriotic tissues, the ERRα OD values were 0.1351 ± 0.0285, while in normal tissues, they were 0.0966 ± 0.03892 (P < 0.0001). The relative OD values for ERβ were 0.0924 ± 0.0223 and 0.0807 ± 0.0243 (P = 0.015). In endometriotic tissues, the OD values for IL-6 were 0.1312 ± 0.0267, while in normal tissues, they were 0.0863 ± 0.0298 (P < 0.0001). As illustrated in Figure 1, the caspase-3 OD values were 0.1196 ± 0.0304 and 0.1416 ± 0.0372 (P = 0.0018), and the caspase-9 values were 0.0699 ± 0.0438 and 0.1127 ± 0.0235 (P < 0.0001).

Effects of PGC-1α regulation on ERRα, ERβ, IL-6, caspase-3, and caspase-9 expression in human endometrial stromal cells

**Figure 2:**
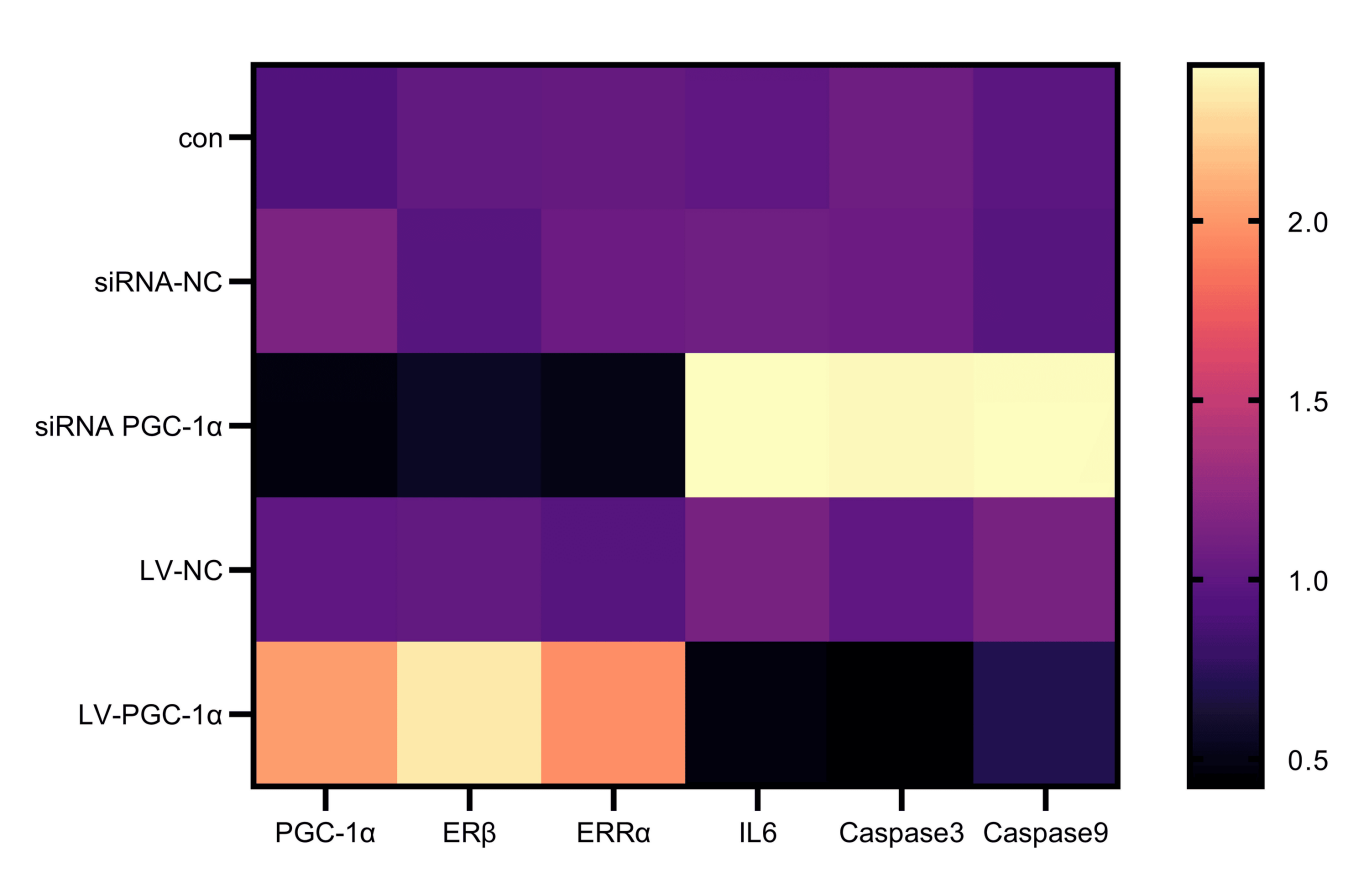
Effects of PGC-1α regulation on the expression of ERRα, ERβ, IL-6, caspase-3, and caspase-9 in human endometrial stromal cells PGC-1α: profiles of peroxisome proliferator-activated receptor-γ coactivator-1α; ERRα: estrogen-related receptor-α; ERβ: estrogen receptor-β; IL-6: interleukin-6; caspase-3: cysteinyl-aspartic acid-specific protease-3; caspase-9: cysteinyl-aspartic acid-specific protease-9; con: group with no treatment; siRNA-NC: group transfected with empty interference plasmid; siRNA PGC-1α: group transfected with PGC-1α interference plasmid; LV-NC: group transfected with empty overexpression plasmid; LV-PGC-1α: group transfected with PGC-1α overexpression plasmid

Expression of PGC-1α, ERRα (P = 0.0024), and ERβ (P = 0.0006) all showed substantial decreases upon transfection with interference plasmids. On the other hand, there was a significant increase in the expression of IL-6 (P = 0.0054), caspase-3 (P = 0.0048), and caspase-9 (P = 0.0047). The expression of PGC-1α, ERRα (P = 0.0245), and ERβ (P < 0.0001) all markedly increased after transfection with overexpression plasmids. There was a significant decrease in the expression of IL-6 (P = 0.0003), caspase-3 (P = 0.0041), and caspase-9 (P = 0.0094), as illustrated in Figure 2.

Effects of PGC-1α regulation on proliferation and apoptosis of endometrial stromal cells

**Figure 3:**
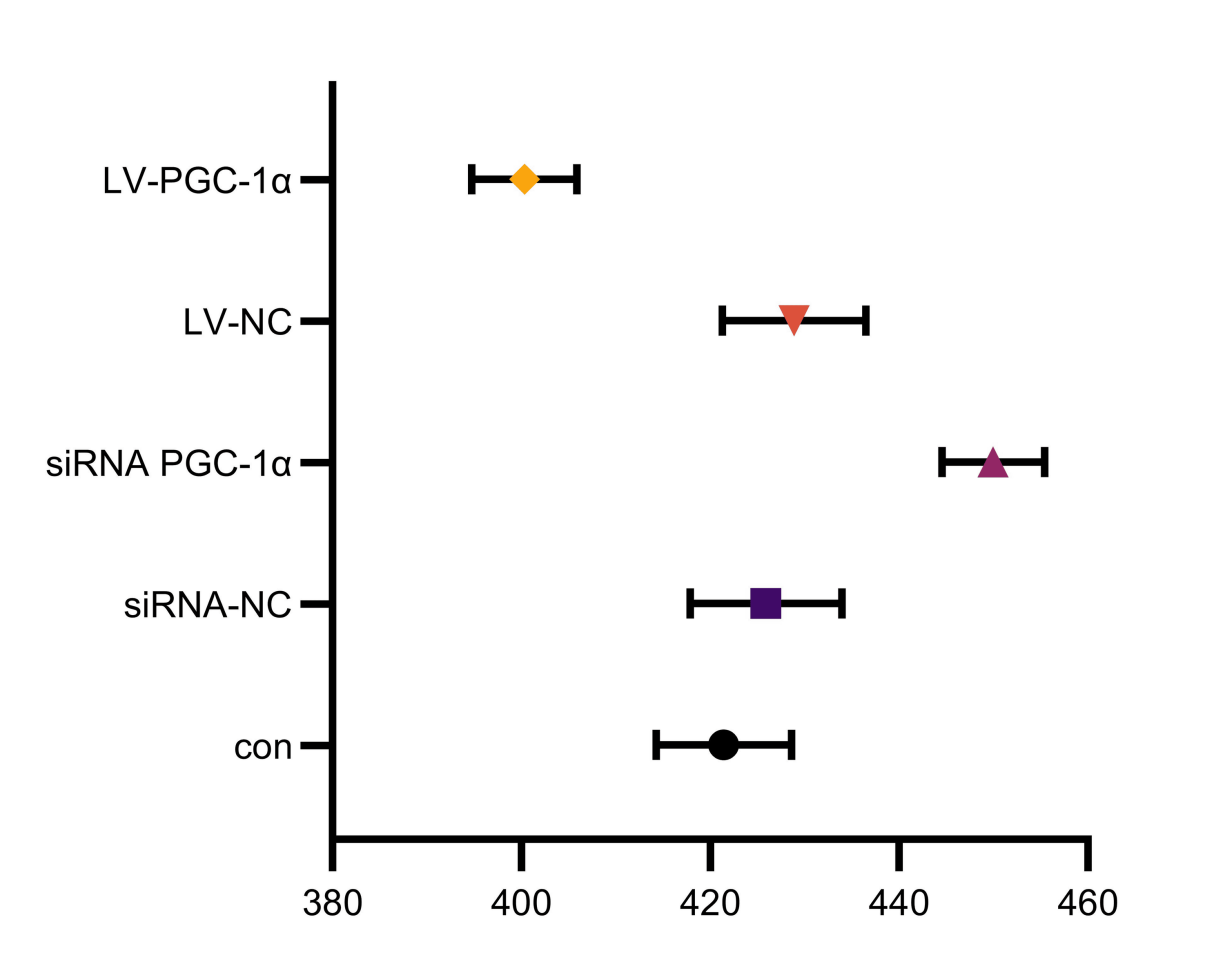
Influence of PGC-1α regulation on the apoptosis of endometrial stromal cells con: group with no treatment; siRNA-NC: group transfected with empty interference plasmid; siRNA PGC-1α: group transfected with PGC-1α interference plasmid; LV-NC: group transfected with empty overexpression plasmid; LV-PGC-1α: group transfected with PGC-1α overexpression plasmid

**Figure 4:**
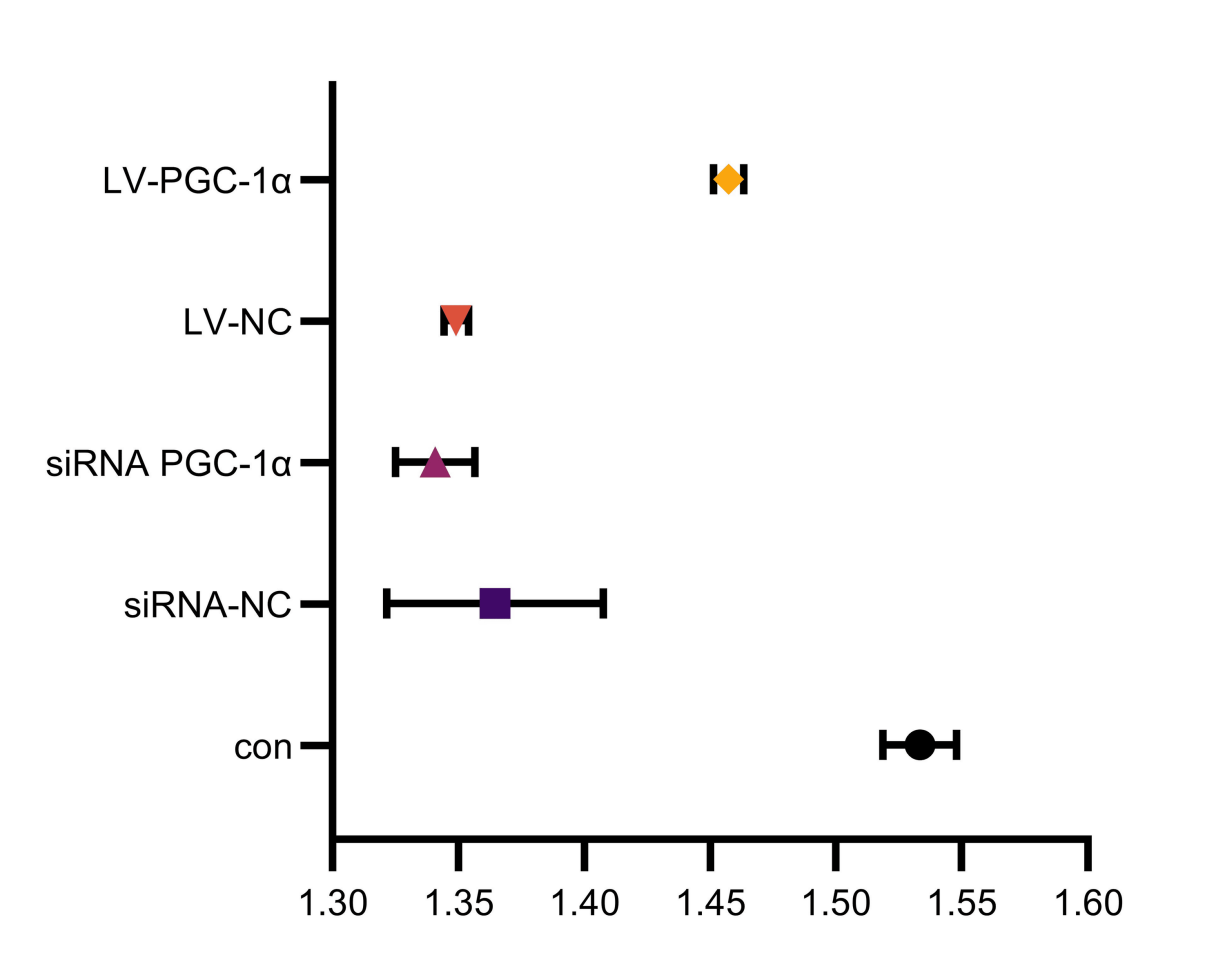
Impact of PGC-1α regulation on the proliferation of endometrial stromal cells con: group with no treatment; siRNA-NC: group transfected with empty interference plasmid; siRNA PGC-1α: group transfected with PGC-1α interference plasmid; LV-NC: group transfected with empty overexpression plasmid; LV-PGC-1α: group transfected with PGC-1α overexpression plasmid

Cell proliferation decreased (not statistically significant, P = 0.4194) and apoptosis increased (P = 0.0127) when PGC-1α expression was silenced. On the other hand, as Figures 3-4 demonstrate, overexpression of PGC-1α decreased apoptosis (P = 0.0064) and increased cell proliferation (P < 0.0001).

## Discussion

The focus of this study was endometriosis, and endometrial stromal cells and endometriosis tissues were thoroughly examined. Within this illness context, the expression variations, interaction dynamics, and effects on cell proliferation and death of molecules like PGC-1α, ERRα, ERβ, IL-6, caspase-3, and caspase-9 were comprehensively examined. As a result, these components' crucial involvement in the pathophysiology of endometriosis was uncovered, giving possible molecular targets for endometriosis therapeutic approaches.

In the first case, a significantly higher expression of PGC-1α was clearly seen in endometrial tissues. PGC-1α functions as a key regulatory hub in cellular energy metabolism and is closely linked to numerous essential biological processes, such as oxidative metabolism and mitochondrial biogenesis. Its increased expression is very likely to upset the intracellular energy metabolic balance, cause oxidative stress, and trigger inflammatory reactions, all of which provide the catalyst for the development and advancement of endometriosis. Using transfection interference plasmid technology to attenuate PGC-1α expression at the cellular experimental level, it was evident that ERRα and ERβ expression levels decreased simultaneously, while IL-6 expression increased and caspase-3 and caspase-9 expressions increased. On the other hand, when overexpression plasmids were transfected to increase the expression of PGC-1α, the expressions of ERRα and ERβ increased, the expression of IL-6 decreased, and the expressions of caspase-3 and caspase-9 simultaneously decreased. These results clearly confirm that PGC-1α is connected to ERRα, ERβ, IL-6, and apoptosis-related variables through a tight and intricate regulatory network. The thorough explanation of PGC-1α's physiological properties by Yanhong and Ruixia [5] has established a strong theoretical foundation for understanding its functional role in the endometriosis environment in the pertinent study field. The central role of PGC-1α in the pathophysiology of endometriosis has been further supported by the idea put forth by Kataoka et al. [6] that the pathway mediated by PGC-1α may be a potential therapeutic target for endometriosis and the review of the research progress of PGC-1α as a key energy metabolism regulator by Wang et al. [7].

The results of this investigation on how PGC-1α affects cell division and death are extremely important. When siRNA technology was used to disrupt PGC-1α expression, cell death was noticeably increased, while cell proliferation showed a declining trend but did not reach a statistically significant level. This event suggests that PGC-1α's control of cell proliferation is not the only factor influencing endometriosis cells; the cell's ability to proliferate is likely maintained by additional compensating regulatory mechanisms or complex signal transduction networks. Unquestionably, PGC-1α has a significant regulatory effect on cell apoptosis, and changes in its expression level can effectively control the expression abundances of components linked to cell apoptosis, which in turn has a significant impact on the state of cell survival. Furthermore, PGC-1α overexpression can significantly increase cell proliferation and suppress apoptosis, which is consistent with tissue-level data and supports the crucial role of PGC-1α in the pathophysiology of endometriosis.

Second, in line with previous related research reports, this study discovered that the expression level of ERβ in endometriotic tissues was significantly higher than that in normal endometrial tissues. By carefully controlling downstream gene expression, ERβ primarily participates in a variety of biological processes, including cell division, proliferation, and apoptosis, which have a significant impact on the regular physiological functioning of endometrial cells. For illustration, Mori et al. [8] delved into the relevant mechanisms of ERβ when probing into the formation of local estrogen and its regulatory role in endometriosis; Bulun et al. [9] accentuated the potential key role of ERβ in the study on the common pathophysiological underpinning of endometriosis and adenomyosis; and Szukiewicz et al. [10] further spotlighted the significance of ERβ in the complex pathological process in the study on the role of epigenetic modifications of endometrial stromal cells and mesenchymal stem cells mediated by estrogen and progesterone in the pathogenesis of endometriosis. Concurrently, as a downstream effector factor of PGC-1α, ERRα also manifested a significant elevation in expression in endometriosis tissues. The in-depth exploration of the two target gene activation pathways of orphan ERR nuclear receptors by Nakadai et al. [11] furnished crucial cues for comprehensively fathoming the role mechanism of ERRα in endometriosis. Li et al. [12] lucidly expounded on its cruciality in energy metabolism regulation through meticulous research on the interaction mechanism between ERRα and ligands by means of molecular dynamics simulation technology. Likhite et al. [13] and Carnesecchi et al. [14] respectively expatiated on the important roles of ERRα in cell proliferation and invasion from disparate research perspectives, further underscoring its indispensability in the endometriosis occurrence and development process.

Additionally, this study showed that patients with endometriosis had a much higher level of IL-6 expression, which is consistent with findings from other similar studies. By encouraging inflammatory reactions and cell proliferation, IL-6 primarily worsens the disease course of endometriosis. The establishment and development of endometriosis can be efficiently accelerated by the abnormally elevated inflammatory response, which can cause endometrial cells to expand and behave invasively. Samimi et al. [15] delved into the mechanism of action of IL-6 while stressing the significant role of inflammation in the pathophysiology of endometriosis; Wenjie et al. [16] further corroborated the importance of IL-6 in the endometriosis pathogenesis through specific research on the expressions of neutrophil to lymphocyte ratio (NLR), C-reactive protein (CRP), IL-6, and IL-17 in endometriosis and their correlations with the disease condition.

Lastly, this study found that caspase-9 expression was greatly reduced, which strongly suggests that the endogenous apoptotic mechanism of endometriosis was significantly hindered. By inhibiting the activation of caspase-3 and caspase-9, the overexpression of PGC-1α may reduce the amount of cell apoptosis and provide favorable conditions for the evolution of endometriosis illness. Bock et al. [17] probed into the crucial roles of caspase-3 and caspase-9 in the cell apoptosis process when expounding on the role mechanism of mitochondria as multifaceted regulators of cell death; Nössing [18] furnished an essential background knowledge framework for understanding its role in endometriosis through the retrospective study on the extensive impact of cell apoptosis in tissue kinetics. The process of cell apoptosis is crucial for preserving the dynamic balance of endometrial cell numbers under normal physiological conditions. On the other hand, aberrant development of endometrial cells outside the uterus and the creation of lesions are caused by the imbalance of cell apoptosis in the endometriosis disease state [19].

The uniqueness of this study, in contrast to previous research, lies in the thorough and methodical examination of the expression alterations and interactions of PGC-1α and its several related signal molecules in endometriosis, as well as the in-depth investigation of their effects on cell division and apoptosis. Prior research often focused on the links between PGC-1α and one or a small number of variables, but this work creatively and thoroughly examined the intricate interactions between PGC-1α and several important molecules, including ERRα, ERβ, IL-6, caspase-3, and caspase-9. This study strategy offers a wider research horizon for the identification of new treatment targets and contributes to a deeper understanding of the endometriosis pathophysiology. The current state of research indicates that no study has been able to analyze the internal interactions of these molecules in endometriosis with such depth and comprehensiveness up to this point.

However, there are certain limitations to this study. First and foremost, the study's sample size is somewhat limited. It is difficult to cover every scenario, and the research depth for some uncommon endometriosis subtypes or cases with unique individual characteristics is still insufficient, even though it may somewhat mimic the expression change trends of PGC-1α and its linked components in endometriosis. Second, the in vivo dynamic change process of PGC-1α in the endometriosis pathogenesis has not been thoroughly and insightfully clarified, and the study mostly focuses on research at the cellular and tissue levels. Considering the aforementioned constraints, future investigations may consider increasing the sample size and, at the same time, using experimental techniques like animal models to fully understand the in vivo mechanism of action of PGC-1α in endometriosis. They may also actively investigate the viability of drug development and treatment plans based on the PGC-1α target. To further improve the effectiveness of endometriosis treatment, a thorough analysis of the intricate regulatory network between these variables will be crucial.

## Conclusions

In summary, this study has systematically investigated the expression profiles of PGC-1α, ERRα, ERβ, IL-6, caspase-3, and caspase-9 in endometriosis tissues and their effects on endometrial stromal cells. The results clearly show that in endometriosis tissue, the expression levels of these molecules are significantly altered compared to normal endometrial tissues. Through cell experiments, we have demonstrated that modulating PGC-1α expression has a profound impact on the expression of related molecules and cell behaviors, such as proliferation and apoptosis. This indicates that PGC-1α potentially plays a crucial role in the pathophysiology of endometriosis by regulating estrogen receptors, inflammatory factors, and apoptotic factors. Our findings provide novel insights and a strong theoretical foundation for the development of endometriosis treatment strategies, filling a knowledge gap in the understanding of the relationship between PGC-1α and key factors in endometriosis.
